# Fructose drives *de novo* lipogenesis affecting metabolic health

**DOI:** 10.1530/JOE-22-0270

**Published:** 2023-03-27

**Authors:** Bettina Geidl-Flueck, Philipp A Gerber

**Affiliations:** 1 Department of Endocrinology, Diabetology and Clinical Nutrition, University Hospital Zurich (USZ) and University of Zurich (UZH), Switzerland

**Keywords:** sugar, glucose, fructose, *de novo* lipogenesis, fatty acids

## Abstract

Despite the existence of numerous studies supporting a pathological link between fructose consumption and the development of the metabolic syndrome and its sequelae, such as non-alcoholic fatty liver disease (NAFLD), this link remains a contentious issue. With this article, we shed a light on the impact of sugar/fructose intake on hepatic *de novo* lipogenesis (DNL), an outcome parameter known to be dysregulated in subjects with type 2 diabetes and/or NAFLD. In this review, we present findings from human intervention studies using physiological doses of sugar as well as mechanistic animal studies. There is evidence from both human and animal studies that fructose is a more potent inducer of hepatic lipogenesis than glucose. This is most likely due to the liver’s prominent physiological role in fructose metabolism, which may be disrupted under pathological conditions by increased hepatic expression of fructolytic and lipogenic enzymes. Increased DNL may not only contribute to ectopic fat deposition (i.e. in the liver), but it may also impair several metabolic processes through DNL-related fatty acids (e.g. beta-cell function, insulin secretion, or insulin sensitivity).

## Introduction

Metabolic health is at risk in societies with an excess supply of energy-dense palatable food and drinks and an everyday life with low physical activity. There is a global epidemic of metabolic syndrome (Saklayen 2018), which includes obesity (particularly visceral adipose tissue accumulation), dyslipidemia, impaired glucose tolerance, and hypertension. Importantly, this syndrome not only affects adults but also children and adolescents, in particular in developing countries (Noubiap *et al.* 2022). Similarly, the prevalence of non-alcoholic fatty liver disease (NAFLD), the hepatic manifestation of the metabolic syndrome, is increasing (Moore 2010, Sahota *et al.* 2020, Riazi *et al.* 2022). The metabolic syndrome, with all of its associated comorbidities, not only burdens the affected individual but also the public health care system (Boudreau *et al.* 2009).

It is commonly acknowledged that an increased body weight, associated with a positive energy balance, is a major trigger for the development of metabolic diseases. It is assumed, however, that factors other than an imbalanced energy intake and expenditure can influence metabolic health. A well-balanced macronutrient intake, characterized by a moderate fat and carbohydrate intake, with a focus on sugar restriction, is regarded as an important component of a healthy diet. A high intake of added sugars, and in particular of fructose – which is often present in a typical western diet – is considered to be a principal factor promoting metabolic derangements (Lim *et al.* 2010, Jensen *et al.* 2018). Despite numerous studies, it is still debated whether the metabolic effects of added sugars are mediated by excess energy intake/weight gain or whether fructose and glucose affect metabolism differently and independently of excess caloric intake. This review aims to shed a light on the current literature regarding this question.

## Sugar consumption and its effects

### Current recommendations

To reduce the risk of developing obesity and metabolic diseases, the World Health Organization recommends that adults and children consume less than 10% (preferably less than 5%) of their energy needs from free sugar (WHO 2015). Importantly, free sugars include monosaccharides and disaccharides added to food and beverages as well as sugars naturally present in honey, syrups, fruit juices, and fruit juice concentrates. Recent studies on sugar intake in Europe, Latin America, and the USA found that mean sugar intakes in most countries were higher than the recommended intake (Fisberg *et al.* 2018, Löwik 2021, DiFrancesco *et al.* 2022). As a consequence, measures to reduce sugar intakes such as better food labeling or taxes on sweetened food are discussed or already implemented in many countries.

### Dietary glucose and fructose

Glucose and fructose are stereoisomers. Fructose displays a higher sweetening power compared to glucose (Moskowitz 1970). Fructose and glucose occur naturally as monosaccharides in fruits and honey but also as sucrose (a disaccharide consisting of glucose and fructose). Other sugar sources include table sugar (sucrose) or high-fructose corn syrup (HFCS) (a mixture of fructose and glucose), concentrated fruit juices, agave or maple syrup, and so on. Sugar added to food and beverages as sweeteners are termed ‘added sugars’. Importantly, the digestion/absorption of sugar from fruits is much slower than that of beverages and thus is unlikely to be associated with any negative effects. Unfavorable metabolic effects are particularly induced by beverages containing high amounts of free sugar that are rapidly absorbed, as detailed below. HFCS is manufactured industrially from corn starch through the isomerization of glucose to fructose. The proportion of fructose varies between 42 and 90% in HFCS (Serna-Saldivar 2016). HFCS with 42% fructose is widely used as a sweetener in processed foods, whereas HFCS with 55% fructose is commonly used in beverage production (Kay Parker 2010). HFCS was first introduced to the market in the USA in the 1970s, and it is now a significant US export product, particularly to developing countries. The average fructose intake increased since the 1970s in the USA (Tappy & Lê 2010). HFCS is a cheap sweetener used in the food and beverage industries, and its consumption is linked to the occurrence of type 2 diabetes (Kmietowicz 2012) and other metabolic diseases, as described below.

### Sugar-sweetened beverage consumption is a risk factor for cardiometabolic diseases

A major source of added sugars are sugar-sweetened beverages (SSBs) (Johnson *et al.* 2009, Malik & Hu 2022). Their consumption has been linked not only to the development of obesity but also to its complications such as type 2 diabetes, NAFLD, and cardiovascular disease (Malik & Hu 2022). Prospective cohort studies from the USA and the UK found an association between high SSB consumption and an increased risk of type 2 diabetes independently of obesity (Imamura *et al.* 2015). Similarly, studies confirmed that habitual SSB consumption is associated with a dose-dependent increase in the risk of dyslipidemia and coronary heart disease (Te Morenga *et al.* 2014, Yin *et al.* 2021). Importantly, studies showed that habitual SSB consumption has a dose-dependent effect on the risk of NAFLD (Ouyang *et al.* 2008, Chen *et al.* 2019) and that SSB intake in early childhood is associated with the later development of hepatic steatosis in adulthood (Sekkarie *et al.* 2021). In addition to metabolic abnormalities, there is evidence of a link between SSB consumption and breast cancer, pancreatic and prostate cancer, and colorectal cancer (Malik & Hu 2022).

Worldwide, SSB intake is still rising (Singh *et al.* 2015, Malik & Hu 2022). However, regional differences regarding SSB consumption are striking. Overall, SSB intake is highest in men and women in Latin America and the Caribbean (average SSB intake about 325 g/day), where it has been rising for decades. In contrast, SSB intake in western high-income countries has stabilized since the 1990s at around 150–200 g/day (Malik & Hu 2022). In Asian countries, SSB consumption is remarkably low (the average intake of SSB is about 30 g/day). Given these data on global SSB consumption, the global burden of obesity and chronic diseases for societies is likely to rise further, particularly in developing countries.

## A specific role for fructose in the etiology of cardiometabolic diseases?

### Differences between fructose and glucose metabolism

Although high sugar consumption is recognized as a risk factor for cardiometabolic diseases, the debate over whether the fructose component of consumed sugar plays a specific role in the etiology of such diseases is still ongoing. This question cannot be easily assessed by epidemiologic studies as fructose is rarely ingested in a pure form but mostly co-ingested with glucose.

**Figure 1:**
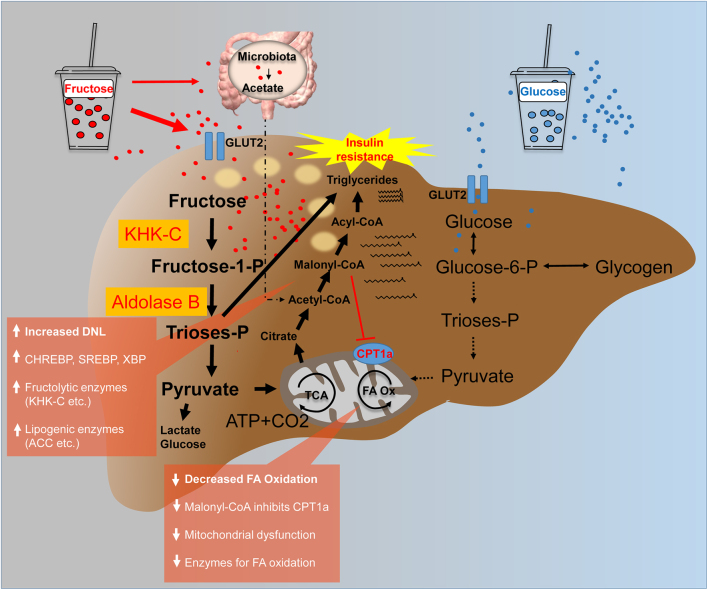
A comparison of the hepatic fructose (left) and glucose (right) metabolism after consumption of high loads of sugar in the form of SSB. It is hypothesized that an increased *de novo* lipogenesis after fructose intake in parallel with a decreased fatty acid oxidation leads to hepatic fat deposition. ACC, acetyl-CoA-carboxylase; ATP, adenosine triphosphate; CPT1a, carnitine palmitoyltransferase 1A; FA, fatty acid; GLUT, glucose transporter; KHK-C, ketohexokinase-C; Ox, oxidation; P, phosphate; SSB, sugar-sweetened beverage; TCA, tricarboxylic acid cycle.

There are important differences regarding the cellular absorption and distribution of glucose and fructose (Maruhama & Macdonald 1973). Fructose is primarily absorbed via facilitated diffusion via glucose transporter 5 (GLUT5) (Burant *et al.* 1992), which is expressed on epithelial intestinal cells, whereas glucose is absorbed via sodium-glucose-cotransporter 1, an active transporter (Gorboulev *et al.* 2012). A proportion of fructose is directly metabolized into glucose in enterocytes. However, when large amounts of fructose are consumed (e.g. when consuming SSB), fructose spills over to the liver and large intestine (Jang *et al.* 2018) (Fig. 1). Fructose and glucose enter the circulation via GLUT5 and GLUT2, respectively (Koepsell 2020). Following that, the liver, which is the primary site of fructose metabolism, extracts a large portion of it (Mendeloff & Weichselbaum 1953). However, it can also be metabolized by the kidney, skeletal muscle, and adipose tissue. Hesley *et al.*(2020) provided a thorough review of tissue-specific fructose metabolism. In contrast, glucose is taken up and metabolized by most mammalian tissues (Thorens & Mueckler 2010). The majority of glucose is taken up by the liver and muscle and stored as glycogen – processes that require insulin. Further amounts of glucose are metabolized by the brain, adipose tissue, and the kidney (Gerich 2000). Following cellular uptake, fructose and glucose are phosphorylated at different rates by specific kinases. Fructokinase is expressed as the two isoforms ketohexokinase-A (KHK-A) and KHK-C. KHK-C is primarily expressed in the liver, but it is also found in the kidney and intestines, whereas KHK-A is more widely expressed (Diggle *et al.* 2009). KHK-C drives hepatic fructose uptake by phosphorylating fructose at a very high rate without feedback inhibition, resulting in a flux of fructose toward the liver (Ishimoto *et al.* 2012) (Fig. 1). Glucose is phosphorylated by glucokinase (GK). Importantly, the phosphorylation rate of KHK is 10 times higher than that of GK. Phosphorylated fructose is cleaved into trioses and enters the glycolytic pathway. Fructose is mainly metabolized into lactic acid and converted to glucose or hepatic glycogen and lipids (Chong *et al.* 2007, Parks *et al.* 2008). Notably, fructose absorption is increased when it is co-ingested with glucose (Rumessen & Gudmand-Høyer 1986). Furthermore, animal studies have shown that consuming high amounts of fructose increases the expression of fructolytic and gluconeogenic enzymes and expands the intestinal cell surface, which improves nutrient absorption (Patel *et al.* 2015*a*, Taylor *et al.* 2021).

### Metabolic effects of regular sugar/fructose intake

Traditionally, easily measurable outcome parameters of known clinical significance (cardiovascular risk markers), such as fasting glucose, insulin, c-peptide, insulin sensitivity/resistance, or serum lipids, are measured for the risk assessment of dietary products regarding metabolic health. However, when metabolic health is defined just as the presence of ideal levels of these markers, fine metabolic changes may be missed. As a result, studies used more subtle outcome parameters to investigate how moderate sugar intake affects the metabolism of healthy men. Indeed, they provide evidence that consumption of SSB containing fructose in moderate amounts leads to metabolic derangements such as decreased hepatic insulin sensitivity (reflected by impaired suppression of glucose production during euglycemic–hyperinsulinemic clamps) (Aeberli *et al.* 2013), induces a shift toward a more atherogenic low-density lipoprotein (LDL) subclass distribution (Aeberli *et al.* 2011) in healthy men, or increases hepatic lipogenic activity (Geidl-Flueck *et al.* 2021).

The latter, an increased *de novo* lipogenesis (DNL), is supposed to be linked to various metabolic complications/perturbations. As a result, the following section focuses on metabolic interactions between dietary sugars, specifically fructose and DNL.

### *De novo* lipogenesis in health and disease

*De novo* lipogenesis (DNL) converts excess dietary carbohydrates (CHO) into fatty acids (FAs). FAs are formed during this process from acetyl-CoA molecules generated directly from CHO catabolism (i.e. glycolysis or fructolysis) or acetate generated by microbiota fructose fermentation (Zhao *et al.* 2020). DNL necessitates the expression of lipogenic pathway enzymes by various cell types, particularly white adipocytes and hepatocytes. DNL contributes to the maintenance of glucose homeostasis. A healthy balance of hepatocyte and adipocyte DNL is essential for maintaining systemic insulin sensitivity (Song *et al.* 2018). The master transcription factors sterol-responsive element-binding protein-1 (SREBP-1) induced by CHO intake/insulin signaling and carbohydrate responsive element-binding protein (ChREBP) stimulated by CHO intake regulate the expression of lipogenic enzymes. DNL provides FA for the structural maintenance of the cells, allows storage of energy from CHO beyond the glycogen store (thus contributing to glucose homeostasis), and regulates FA oxidation.

The process of FA synthesis in the liver has been identified as being of particular interest in the etiology of the metabolic syndrome as well as a specific feature of NAFLD (Donnelly *et al.* 2005, Lambert *et al.* 2014, Imamura *et al.* 2020). Clinical studies showed that DNL is increased in subjects with increased hepatic fat content (isotope approaches) (Diraison *et al.* 2003, Lambert *et al.* 2014). Furthermore, DNL was found to be positively related to intrahepatic triglyceride (TAG) levels (Diraison *et al.* 2003, Lambert *et al.* 2014) and negatively related to hepatic and whole-body insulin sensitivity (Smith *et al.* 2020). DNL is supposed to increase intrahepatic fat both by providing FA for TAG synthesis and by inhibiting FA oxidation promoting the re-esterification process. Importantly, accumulating intermediates (i.e. malonyl-CoA) inhibit FA import into the mitochondria and thus FA oxidation (McGarry *et al.* 1977, Cox *et al.* 2012). Furthermore, a clinical study (crossover) showed that an increase in DNL induced by a diet high in simple sugars correlates with triglyceridemia both in lean and in obese subjects (Hudgins *et al.* 2000). In addition, increased concentrations of DNL-related FAs (i.e. palmitate 16:0) have been linked to the metabolic syndrome in observational and interventional studies (Vessby 2003). Mechanistic *in vitro* studies suggest that palmitate impairs beta-cell function via ceramide formation, causing endoplasmic reticulum stress, and induces the apoptotic mitochondrial pathway (Maedler *et al.* 2001, Maedler *et al.* 2003, Cunha *et al.* 2008). Other studies revealed that palmitate stimulates interleukin-6 expression, a mechanism involved in the pathogenesis of insulin resistance and vascular inflammation (Rotter *et al.* 2003, Staiger *et al.* 2004, Weigert *et al.* 2004, Testa *et al.* 2006, Korbecki & Bajdak-Rusinek 2019). Therefore, from a clinical perspective, DNL may serve as a valuable marker for the development of cardiometabolic disease beyond hepatic lipid accumulation/NAFLD.

### The impact of macronutrients on DNL – insights from human intervention studies

Regarding the question of how different macronutrients impact metabolic health, early human studies compared the effects of diets with different carbohydrate and fat intake on metabolic outcomes. Later, the effects of different forms of carbohydrates were compared (e.g. simple sugars vs complex carbohydrates or different types of sugar) in studies with children or adults, with or without obesity/metabolic disease. Interventions aimed at increasing sugar/fructose consumption, e.g. by SSB intake or decreasing sugar/fructose intake by prescription of sugar/fructose restriction (Donnelly *et al.* 2005, Lambert *et al.* 2014). Finally, they all contribute to the understanding of the relationship between CHO intake and metabolic complications in general as well as the relative importance of fructose and glucose. Importantly, studies on the effects of sugar consumption on DNL are rarely comparable due to significant differences in the study populations, interventions, and/or methods used. (Studies discussed below are summarized in Table 1).

**Table 1:** Overview of studies measuring the effects of dietary interventions on hepatic DNL by tracer methodology.

Intervention	Duration	Subjects	*N*	DNL measurement	Result	Reference
Eucaloric liquid formula diets –Low-fat diet (10% of calories as fat and 75% as glucose polymers) –High-fat diet (40% of calories as fat and 45% as glucose polymers)	25 days	Healthy men and women Younger adults Normal weight	10	Postprandial DNL labeling of palmitate with 13C-acetate, MIDA; linoleate dilution method	Dietary substitution of carbohydrate (CHO) for fat stimulates the hepatic fatty acid synthesis	Hudgins *et al.* (1996)
Isocaloric diets with the same macronutrient composition –High-fructose diet (25% caloric intake; beverage) –Complex CHO (solid) diet (replaced fructose)	9 days	Healthy men All age groups Normal weight	8	Postprandial DNL Labeling of palmitate with 13C-acetate, MIDA	High-fructose diet is associated with higher hepatic DNL	Schwarz *et al.* (2015)
Daily SSB consumption (25% of required caloric intake provided as SSB; 8-week outpatient intervention with* ad libitum* diet, 2-week energy-balanced inpatient intervention) –Glucose-SSB –Fructose-SSB	10 weeks	Men and women Middle-aged Overweight/obese	32	Postprandial DNL Labeling of palmitate with 13C-acetate, MIDA	High fructose increases hepatic DNL	Stanhope *et al.* (2009)
Beverage consumption containing glucose (GLC) and/or fructose (FRC) –100:0 GLC:FRC –50:50 GLC:FRC –25:75 GLC:FRC	Single exposure	Healthy men and women Younger adults Normal weight	6	Postprandial DNL Labeling of palmitate with 13C-acetate, MIDA	Acute intake of fructose stimulates hepatic lipogenesis	Parks *et al.* (2008)
Daily SSB (3×0.2 L SSB/day equivalent to 80g sugar intake/day) consumption or SSB abstinence –Glucose–SSB –Fructose–SSB –Sucrose–SSB	6 weeks	Healthy men Younger adults Normal weight	94	Basal DNL Labeling of palmitate with 13C-acetate, MIDA	Fructose and sucrose increase basal hepatic lipogenic activity	Geidl-Flueck *et al.* (2021)
Dietary sugar restriction –Low free sugar diet –‘Usual’ diet	8 weeks	Obese boys with NAFLD	29	Labeling of palmitate with 2H_2_O, MIDA	Dietary sugar restriction reduces hepatic DNL	Cohen *et al.* (2021)
Isocaloric fructose restriction –Starch substituted for sugar (reduced caloric intake from fructose from 12% to 4% of total energy intake)	9 days	Children (male and female) with obesity and metabolic syndrome and habitual high sugar consumption (fructose intake >50 g/day)	41	Postprandial DNL Labeling of palmitate with 13C-acetate, MIDA	Isocaloric fructose restriction decreases hepatic DNL	Schwarz *et al.* (2017)

Of note, the process of hepatic DNL is assessed by applying different methods that all analyze FA bound to very low-density lipoproteins (VLDL). They range from calculating FA desaturation indices to calculating the percentage of surrogate FA for newly formed FA (i.e. palmitate) in total FA to labeling newly formed FA with isotopes to calculate fractional DNL or fractional secretion rates of *de novo* synthesized FAs (Hellerstein *et al.* 1991). Measurement of DNL by isotope labeling methodology is considered the gold standard. However, it is costly and thus only appropriate for studies with small sample sizes.

Initially, it was assessed by Hudgins *et al.* how the fat and CHO content of a diet impacts hepatic DNL in healthy men. Subjects were randomly assigned to either an eucaloric liquid high-fat diet (40% of calories as fat and 45% as glucose polymers, *n* = 3) or a high-CHO diet (10% of calories as fat and 75% as glucose polymers, *n* = 7) for 25 days. DNL was increased in men on a high-CHO diet after 10 days, reflected as palmitate-enriched, linoleate-deficient VLDL triglycerides, and palmitate synthesis (mass isotopomer distribution analysis (MIDA) of palmitate labeled with 13C-acetate) was increased after 25 days compared to the high-fat diet (Hudgins *et al.* 1996).

In a later study, Schwarz *et al.* (2015) compared the effects of a high-fructose (25% energy content), weight-maintenance diet to those of an isocaloric diet with the same macronutrient distribution but complex carbohydrates (CCHO) substituted for fructose (crossover design, *n* = 8). Importantly, fructose was provided as beverages, whereas complex carbohydrates were provided as solid food. After 9 days of intervention, high-fructose intake was associated with higher fractional hepatic DNL (MIDA of palmitate labeled with 13C-acetate) compared to the diet in which fructose was replaced by CCHO (Schwarz *et al.* 2015). Stanhope *et al.* (2009) investigated the effects of glucose and fructose consumption on hepatic DNL in obese subjects after 10 weeks of consumption of glucose- or fructose-sweetened beverages providing 25% of energy requirements. Postprandial DNL was increased after fructose consumption (MIDA of palmitate labeled with 13C-acetate) (Stanhope *et al.* 2009).

The effects of different hexoses on hepatic DNL were investigated by Parks *et al.* (2008). Healthy subjects (*n* = 6) were challenged with sweetened beverages (85 g sugar) containing pure glucose (100:0) or mixtures of fructose and glucose (50:50 or 75:25) on three separate occasions in a random and blinded order. The beverages containing fructose stimulated DNL more potently compared with the beverages containing pure glucose (MIDA of palmitate labeled with 13C-acetate) (Parks *et al.* 2008).

Aside from the postprandial effect of fructose consumption on DNL which has been studied extensively, the effect of regular fructose consumption on basal hepatic lipogenic activity is of interest. Formation of new FAs requires both the expression of lipogenic enzymes and the availability of substrate (acetyl-CoA). FA synthesis, as measured by a constant infusion of glucose (as a substrate for FA synthesis) and 13C-acetate, reflects hepatic lipogenic activity, which is determined by lipogenic enzyme expression. Thus, in such a setting, differences regarding absorption rates of different sugar types do not influence the measurement. The effect of daily SSB consumption on liver lipogenic activity was studied in 94 healthy men by providing daily glucose, fructose, or sucrose-containing drinks (3×0.2 L SSB/day resulting in a sugar intake of 80g/day) in a randomized way during 6 weeks. The study with SSB consumption in a close to real-life setting showed that fructose and sucrose, but not glucose, increased the basal lipogenic activity of the liver (MIDA of palmitate labeled with 13C-acetate) (*n* = 94, randomized controlled trial (RCT)) as compared to a control group. This is most likely due to fructose-containing beverages causing an increase in the expression of lipogenic genes in the liver (Geidl-Flueck *et al.* 2021).

Further studies assessed and clarified the role of DNL in fructose-induced hypertriglyceridemia and whether physical activity prevents hypertriglyceridemia. Egli *et al.* examined healthy subjects (*n* = 8) after 4 days of either a weight-maintaining low-fructose diet (control), a high-fructose diet with low physical activity, or a high-fructose diet with high physical activity. Fasting and postprandial TAG and 13C-palmitate in triglyceride-rich lipoproteins were increased after a high-fructose diet compared to control after an oral challenge with 13C-fructose. Those parameters remained unchanged after the high-fructose/high physical activity intervention, indicating that sport protects against fructose-induced triglyceridemia. The underlying mechanism induced by physical activity (i.e. reduced DNL from fructose or improved TAG clearance) was not resolved by this study. The same authors also tested the hypothesis that exercise prevents a fructose-induced rise in VLDL triglycerides (VLDL-TGs) by decreasing fructose conversion into glucose and VLDL-TGs and fructose carbon storage into hepatic glycogen and lipids (Egli *et al.* 2016). Eight healthy men were placed on a weight-maintenance high-fructose diet (SSB) for 4 days before the metabolic fate of 13C-labeled fructose with or without physical activity was investigated. Exercise increased fructose oxidation. However, it did not abolish fructose conversion into glucose or did not prevent DNL (AUC of VLDL-13C palmitate). These findings imply that fructose-induced DNL occurs regardless of the degree of saturation of other fructose metabolism pathways.

So far, studies that assessed the effect of increased CHO/sugar/fructose consumption on DNL were discussed. Overall, findings from various clinical studies indicate that carbohydrates, particularly when consumed as simple sugars and in liquid form, promote hepatic lipogenesis even when maintenance dietary interventions are used. Furthermore, studies using fructose and glucose interventions revealed that fructose is a more potent inducer of hepatic lipogenesis than glucose.

In addition to these findings, some studies deal with the question of how a reduction/restriction of sugar/fructose consumption impacts DNL.

There is evidence that a general dietary sugar restriction (which also leads to a reduction in fructose intake) results in lower DNL. A link between free sugar consumption and DNL was confirmed by Cohen *et al.* (2021) who conducted a trial with adolescent boys suffering from NAFLD. A low-sugar diet for 8 weeks reduced DNL (and hepatic fat content) compared to their usual diet, as measured by a lower percentage of newly synthesized palmitate in plasma TAG (labeled with deuterated 2H_2_O) (Cohen *et al.* 2021) (*n* = 29, RCT). Similarly, Schwarz *et al.* (2017) demonstrated in a study with obese children that restricting sugar/fructose intake for 9 days reduced hepatic DNL (fractional DNL after a test meal containing 13C-acetate) (*n* = 41). In this study, dietary sugars were substituted by complex carbohydrates.

Both intervention studies that increased sugar/fructose intake and those that reduced fructose intake provide evidence that sugar/fructose intake influences hepatic DNL. Importantly, the few studies that specifically assessed the effects of different hexoses (i.e. glucose and fructose) support the hypothesis that fructose is a more potent inducer of lipogenesis than glucose (Parks *et al.* 2008, Geidl-Flueck *et al.* 2021).

## Fructose vs glucose metabolism – mechanistic insights from animal studies

Insights into mechanisms underlying the differences in glucose and fructose metabolism were gained from animal studies (Maruhama & Macdonald 1973, Geidl-Flueck & Gerber 2017). Several important transcription factors control carbohydrate metabolism. We focus on the role of ChREBP (Yamashita *et al.* 2001) and SREBP-1 (Wang *et al.* 1994) in the regulation of CHO flux. They regulate glycolytic and fructolytic gene expression, as well as the expression of lipogenic genes. Glucose and fructose, to varying degrees, stimulate their expression and activity. Importantly, the expression of both transcription factors is increased in the livers of NAFLD patients (Kohjima *et al.* 2007, Benhamed *et al.* 2012).

ChREBP is most strongly expressed in the liver, white and brown adipose tissue, and also the small intestine and muscle (Iizuka *et al.* 2004). Lipogenic enzyme expression is reduced in mice with a genetic deletion of the ChREBP transcription factor (Iizuka *et al.* 2004). They display an impaired glucose tolerance as a consequence of reduced glucose disposal. ChREBP deletion shifts the flux from excess CHO to glycogen storage. It increases glycogen content in the liver and reduces the hepatic fat content. ChREBP-knockout animals are fructose intolerant due to decreased expression of fructolytic and lipogenic enzymes, resulting in death when fed high-sugar diets. Liver-specific knockout of ChREBP in mice (L-ChREBP^–^^/–^) results in reduced SREBP1c at RNA and protein levels, suggesting that both transcription factors coordinately regulate lipogenic gene expression (Linden *et al.* 2018).

Feeding studies revealed that fructose induces hepatic ChREBP and its targets more potently than glucose (Koo *et al.* 2009, Kim *et al.* 2016, Softic *et al.* 2016, Softic *et al.* 2017). Further, it is also activated by glycerol that is generated during fructolysis. As a result, ChREBP activation is thought to be related to hexose- and triose-phosphate levels (Kim *et al.* 2016).

SREBP is expressed in different isoforms. SREBP-1c induces lipogenic gene expression in response to carbohydrate feeding. SREBP1c mRNA expression is regulated by the TOR signaling pathway and the insulin signaling pathway. For full induction of SREBP-1c expression as well as for its translocation to the nucleus, hepatic insulin signaling is required (Haas *et al.* 2012). In mice, a high-fructose diet induces SREBP-1c expression more potently than a standard chow diet.

Furthermore, mechanistic studies provided evidence that fructose reduces hepatic FA oxidation by different mechanisms. One early *in vitro* study found that fructose, as a competing substrate for oxidation, inhibits long-chain FA oxidation (Prager & Ontko 1976). A further study showed that fructose feeding reduces the expression of peroxisome proliferator-activated receptor and FA oxidation enzymes (Nagai *et al.* 2002). Furthermore, fructose feeding raises malonyl-CoA levels (which inhibits transport of FA by CPT1a into the mitochondria), causes mitochondrial dysfunction (reduced mitochondrial size and protein mass, specifically FA oxidation pathway proteins and CPT1a levels), and increases acetylation of mitochondrial proteins in mice (Softic *et al.* 2019).

The levels of expression of fructolytic pathway enzymes determine the relative contribution of tissues to fructose metabolism. KHK-C is considered to be a key enzyme in fructose metabolism phosphorylating fructose at a high rate as described above. KHK-C is highly expressed in hepatocytes (Diggle *et al.* 2009), but it is also found in the intestine, adipose tissue, kidney, and pancreas (Ishimoto *et al.* 2012). KHK-C knockout mice fail to metabolize fructose, leading to high-fructose concentrations in the blood and urine (Patel *et al.* 2015*b*). Both KHK-C deletion and KHK-C blockade protect against fructose-induced metabolic perturbations (Patel *et al.* 2015*b*, Lanaspa *et al.* 2018, Softic *et al.* 2019). Deletion of the KHK-A isoform exacerbates fructose-induced metabolic syndrome probably due to an increased fructose supply to the liver (Ishimoto *et al.* 2012).

Clinical studies show that patients with NAFLD have increased expression of KHK-C in the liver (Ouyang *et al.* 2008) and that inhibiting KHK-C reduces liver fat in NAFLD (Kazierad *et al.* 2021).

## Possible mechanisms by which sugar/fructose consumption impacts fat distribution/deposition

Ectopic fat deposition is linked to metabolic syndrome and NAFLD and is thought to be exacerbated by a high sugar intake (Ma *et al.* 2016). It is suggested that lipid deposition is promoted by CHO-induced DNL that reduces FA oxidation and by alterations of FA flux. A meta-analysis of randomized controlled trials demonstrated that high-sugar (fructose or sucrose) hypercaloric diets increased liver and muscle fat in comparison to eucaloric control diets (Ma *et al.* 2016). Of course, data from studies that used ‘close to real-life interventions’ with high but not excessive sugar intake would provide the most relevant information about the effects of sugar consumption on fat distribution in individuals. A study by Maerks *et al.* compared the effects of SSB containing sucrose to those of isocaloric milk and a non-caloric soft drink (one liter of drink/day for 6 months) on ectopic fat deposition. Consumption of sucrose-containing SSB for 6 months increases not only hepatic fat content but also muscle and visceral fat in obese subjects, whereas no such effects were observed in the other groups (Maersk *et al.* 2012). However, studies that specifically compare the impact of different types of sugars on fat distribution are scarce (Lecoultre *et al.* 2013). Stanhope *et al.* compared the effects of fructose and glucose-sweetened beverages on body fat distribution in subjects with obesity by quantification of subcutaneous, visceral, and abdominal fat. Consumption of fructose- but not glucose-sweetened beverages (providing 25% of energy requirements) for 10 weeks significantly increased visceral abdominal fat (Stanhope *et al.* 2009). In contrast, glucose consumption increased subcutaneous fat. Data about a fat deposition in the liver and muscle were not collected. In a later study, Schwarz *et al.* used magnetic resonance spectroscopy to investigate the effects of a high-fructose weight-maintenance diet on liver fat. They discovered that 9 days of a high-fructose diet (25% energy content) increased both liver fat and DNL (Schwarz *et al.* 2015). Different mechanisms underlying fat deposition have been suggested that implicate fructose. It is hypothesized that fructose consumption reduces FA oxidation more than glucose consumption and that fructose consumption raises cortisol levels, promoting visceral adiposity and/or lipid deposition in the liver. Cox *et al.* investigated the effects of SSB consumption on substrate utilization and energy expenditure in subjects with obesity. They found that the intake of fructose, but not glucose, reduced resting energy expenditure and postprandial fat oxidation while increasing postprandial carbohydrate oxidation. This finding suggests that lipid deposition may result from sparing FA from oxidation. DiNicolantonio *et al.* proposed that fructose plays a specific role in visceral fat deposition via glucocorticoid-mediated mechanisms (DiNicolantonio *et al.* 2018). Visceral fat is known to accumulate under pathological conditions where cortisol levels are increased, such as Cushing’s syndrome. Fructose consumption is thought to raise cortisol levels by promoting inflammatory processes in adipose tissue and stimulating the hypothalamus, resulting in the release of corticotropin-releasing factor. Cortisol increases the flux of FA from subcutaneous adipose tissue to visceral fat depots, impairing organ function (DiNicolantonio *et al.* 2018) and leading to an unfavorable fat distribution in lean individuals, i.e. a body shape described as thin outside, fat inside, which is associated with an increased risk for the metabolic syndrome (DiNicolantonio *et al.* 2018). Taken together, studies provide evidence that fructose and sucrose consumption promote ectopic fat deposition associated with an increased risk for metabolic disease and cardiovascular events (Gruzdeva *et al.* 2018). This is most likely due to a simultaneous increase in DNL and decrease in FA oxidation, but it could also be due to increased FA flux from subcutaneous adipose tissue to other tissues (visceral fat and the liver).

## Conclusions

A high intake of free sugar as SSB increases the risk of obesity, cardiometabolic diseases, and NAFLD. A central role must be attributed to fructose in the development of these diseases. It is not only a strong inducer of DNL, but it is also a known cause of ectopic fat deposition by reducing fat oxidation and increasing FA flux to visceral fat and the liver. Most importantly, fructose-specific effects occur independently from overfeeding in healthy subjects. There are several mechanisms by which high-fructose consumers increase fructose absorption and catabolism in the liver, exacerbating the metabolic effects. Sugar/fructose consumption should be reduced to avoid these unfavorable metabolic adaptations.

## Declaration of interest

The authors declare no conflict of interests regarding this work.

## Funding

This work was supported by the Heuberg foundation.

## Author contribution statement

Bettina Geidl-Flueck and Philipp Gerber wrote and revised the manuscript.

## References

[bib1] Aeberli I Gerber PA Hochuli M Kohler S Haile SR Gouni-Berthold I Berthold HK Spinas GA Berneis K 2011 Low to moderate sugar-sweetened beverage consumption impairs glucose and lipid metabolism and promotes inflammation in healthy young men: a randomized controlled trial. American Journal of Clinical Nutrition 94 479–485. (10.3945/ajcn.111.013540) 21677052

[bib2] Aeberli I Hochuli M Gerber PA Sze L Murer SB Tappy L Spinas GA Berneis K 2013 Moderate amounts of fructose consumption impair insulin sensitivity in healthy young men: a randomized controlled trial. Diabetes Care 36 150–156. (10.2337/dc12-0540) 22933433 PMC3526231

[bib3] Benhamed F Denechaud PD Lemoine M Robichon C Moldes M Bertrand-Michel J Ratziu V Serfaty L Housset C Capeau J 2012 The lipogenic transcription factor ChREBP dissociates hepatic steatosis from insulin resistance in mice and humans. Journal of Clinical Investigation 122 2176–2194. (10.1172/JCI41636) 22546860 PMC3366390

[bib4] Boudreau DM Malone DC Raebel MA Fishman PA Nichols GA Feldstein AC Boscoe AN Ben-Joseph RH Magid DJ Okamoto LJ 2009 Health care utilization and costs by metabolic syndrome risk factors. Metabolic Syndrome and Related Disorders 7 305–314. (10.1089/met.2008.0070) 19558267

[bib5] Burant CF Takeda J Brot-Laroche E Bell GI Davidson NO 1992 Fructose transporter in human spermatozoa and small intestine is GLUT5. Journal of Biological Chemistry 267 14523–14526. (10.1016/S0021-9258(1842067-4) 1634504

[bib6] Chen H Wang J Li Z Lam CWK Xiao Y Wu Q & Zhang W 2019 Consumption of sugar-sweetened beverages has a dose-dependent effect on the risk of non-alcoholic fatty liver disease: an updated systematic review and dose-response meta-analysis. International Journal of Environmental Research and Public Health 16 2192. (10.3390/ijerph16122192) PMC6617076 31234281

[bib7] Chong MF Fielding BA Frayn KN 2007 Mechanisms for the acute effect of fructose on postprandial lipemia. American Journal of Clinical Nutrition 85 1511–1520. (10.1093/ajcn/85.6.1511) 17556686

[bib8] Cohen CC Li KW Alazraki AL Beysen C Carrier CA Cleeton RL Dandan M Figueroa J Knight-Scott J Knott CJ 2021 Dietary sugar restriction reduces hepatic de novo lipogenesis in adolescent boys with fatty liver disease. Journal of Clinical Investigation 131 e150996. (10.1172/JCI150996) PMC8670836 34907907

[bib9] Cox CL Stanhope KL Schwarz JM Graham JL Hatcher B Griffen SC Bremer AA Berglund L McGahan JP Havel PJ 2012 Consumption of fructose-sweetened beverages for 10 weeks reduces net fat oxidation and energy expenditure in overweight/obese men and women. European Journal of Clinical Nutrition 66 201–208. (10.1038/ejcn.2011.159) 21952692 PMC3252467

[bib10] Cunha DA Hekerman P Ladrière L Bazarra-Castro A Ortis F Wakeham MC Moore F Rasschaert J Cardozo AK Bellomo E 2008 Initiation and execution of lipotoxic ER stress in pancreatic beta-cells. Journal of Cell Science 121 2308–2318. (10.1242/jcs.026062) 18559892 PMC3675788

[bib11] DiFrancesco L Fulgoni VL Gaine PC Scott MO & Ricciuto L 2022 Trends in added sugars intake and sources among U.S. adults using the National Health and Nutrition Examination Survey (NHANES) 2001–2018. Frontiers in Nutrition 9 897952. (10.3389/fnut.2022.897952**)** 36061886 PMC9434277

[bib12] Diggle CP Shires M Leitch D Brooke D Carr IM Markham AF Hayward BE Asipu A & Bonthron DT 2009 Ketohexokinase: expression and localization of the principal fructose-metabolizing enzyme. Journal of Histochemistry and Cytochemistry 57 763–774. (10.1369/jhc.2009.953190**)** 19365088 PMC2713076

[bib13] DiNicolantonio JJ Mehta V Onkaramurthy N O'Keefe JH 2018 Fructose-induced inflammation and increased cortisol: a new mechanism for how sugar induces visceral adiposity. Progress in Cardiovascular Diseases 61 3–9. (10.1016/j.pcad.2017.12.001) 29225114

[bib14] Diraison F Moulin P Beylot M 2003 Contribution of hepatic de novo lipogenesis and reesterification of plasma non esterified fatty acids to plasma triglyceride synthesis during non-alcoholic fatty liver disease. Diabetes and Metabolism 29 478–485. (10.1016/s1262-3636(0770061-7) 14631324

[bib15] Donnelly KL Smith CI Schwarzenberg SJ Jessurun J Boldt MD Parks EJ 2005 Sources of fatty acids stored in liver and secreted via lipoproteins in patients with nonalcoholic fatty liver disease. Journal of Clinical Investigation 115 1343–1351. (10.1172/JCI23621) 15864352 PMC1087172

[bib16] Egli L Lecoultre V Cros J Rosset R Marques AS Schneiter P Hodson L Gabert L Laville M Tappy L 2016 Exercise performed immediately after fructose ingestion enhances fructose oxidation and suppresses fructose storage1. American Journal of Clinical Nutrition 103 348–355. (10.3945/ajcn.115.116988) 26702120

[bib17] Fisberg M Kovalskys I Gómez G Rigotti A Sanabria LYC García MCY Torres RGP Herrera-Cuenca M Zimberg IZ Koletzko B 2018 Total and added sugar intake: assessment in eight Latin American countries. Nutrients 10 389. (10.3390/nu10040389) PMC5946174 29565308

[bib18] Geidl-Flueck B & Gerber PA 2017 Insights into the hexose liver metabolism-glucose versus fructose. Nutrients 9 1026. (10.3390/nu9091026) PMC5622786 28926951

[bib19] Geidl-Flueck B Hochuli M Németh Á Eberl A Derron N Köfeler HC Tappy L Berneis K Spinas GA Gerber PA 2021 Fructose- and sucrose- but not glucose-sweetened beverages promote hepatic de novo lipogenesis: a randomized controlled trial. Journal of Hepatology 75 46–54. (10.1016/j.jhep.2021.02.027) 33684506

[bib20] Gerich JE 2000 Physiology of glucose homeostasis. Diabetes, Obesity and Metabolism 2 345–350. (10.1046/j.1463-1326.2000.00085.x) 11225963

[bib21] Gorboulev V Schürmann A Vallon V Kipp H Jaschke A Klessen D Friedrich A Scherneck S Rieg T Cunard R 2012 Na(+)-D-glucose cotransporter SGLT1 is pivotal for intestinal glucose absorption and glucose-dependent incretin secretion. Diabetes 61 187–196. (10.2337/db11-1029) 22124465 PMC3237647

[bib22] Gruzdeva O Borodkina D Uchasova E Dyleva Y Barbarash O 2018 Localization of fat depots and cardiovascular risk. Lipids in Health and Disease 17 218. (10.1186/s12944-018-0856-8) PMC6138918 30219068

[bib23] Haas JT Miao J Chanda D Wang Y Zhao E Haas ME Hirschey M Vaitheesvaran B Farese RV Jr Kurland IJ 2012 Hepatic insulin signaling is required for obesity-dependent expression of SREBP-1c mRNA but not for feeding-dependent expression. Cell Metabolism 15 873–884. (10.1016/j.cmet.2012.05.002) 22682225 PMC3383842

[bib24] Hellerstein MK Christiansen M Kaempfer S Kletke C Wu K Reid JS Mulligan K Hellerstein NS Shackleton CH 1991 Measurement of de novo hepatic lipogenesis in humans using stable isotopes. Journal of Clinical Investigation 87 1841–1852. (10.1172/JCI115206) 2022750 PMC295308

[bib25] Helsley RN Moreau F Gupta MK Radulescu A DeBosch B Softic S 2020 Tissue-specific fructose metabolism in obesity and diabetes. Current Diabetes Reports 20 64. (10.1007/s11892-020-01342-8) PMC10208418 33057854

[bib26] Hudgins LC Hellerstein M Seidman C Neese R Diakun J Hirsch J 1996 Human fatty acid synthesis is stimulated by a eucaloric low fat, high carbohydrate diet. Journal of Clinical Investigation 97 2081–2091. (10.1172/JCI118645) 8621798 PMC507283

[bib27] Hudgins LC Hellerstein MK Seidman CE Neese RA Tremaroli JD Hirsch J 2000 Relationship between carbohydrate-induced hypertriglyceridemia and fatty acid synthesis in lean and obese subjects. Journal of Lipid Research 41 595–604. (10.1016/S0022-2275(2032407-X) 10744780

[bib28] Iizuka K Bruick RK Liang G Horton JD & Uyeda K 2004 Deficiency of carbohydrate response element-binding protein (ChREBP) reduces lipogenesis as well as glycolysis. PNAS 101 7281–7286. (10.1073/pnas.0401516101) 15118080 PMC409910

[bib29] Imamura F O'Connor L Ye Z Mursu J Hayashino Y Bhupathiraju SN Forouhi NG 2015 Consumption of sugar sweetened beverages, artificially sweetened beverages, and fruit juice and incidence of type 2 diabetes: systematic review, meta-analysis, and estimation of population attributable fraction. BMJ 351 h3576. (10.1136/bmj.h3576) PMC4510779 26199070

[bib30] Imamura F Fretts AM Marklund M Ardisson Korat AV Yang WS Lankinen M Qureshi W Helmer C Chen TA Virtanen JK 2020 Fatty acids in the de novo lipogenesis pathway and incidence of type 2 diabetes: A pooled analysis of prospective cohort studies. PLOS Medicine 17 e1003102. (10.1371/journal.pmed.1003102) PMC7292352 32530938

[bib31] Ishimoto T Lanaspa MA Le MT Garcia GE Diggle CP Maclean PS Jackman MR Asipu A Roncal-Jimenez CA Kosugi T 2012 Opposing effects of fructokinase C and A isoforms on fructose-induced metabolic syndrome in mice. PNAS 109 4320–4325. (10.1073/pnas.1119908109) 22371574 PMC3306692

[bib32] Jang C Hui S Lu W Cowan AJ Morscher RJ Lee G Liu W Tesz GJ Birnbaum MJ Rabinowitz JD 2018 The small intestine converts dietary fructose into glucose and organic acids. Cell Metabolism 27 351–361.e3. (10.1016/j.cmet.2017.12.016) 29414685 PMC6032988

[bib33] Jensen T Abdelmalek MF Sullivan S Nadeau KJ Green M Roncal C Nakagawa T Kuwabara M Sato Y Kang DH 2018 Fructose and sugar: a major mediator of non-alcoholic fatty liver disease. Journal of Hepatology 68 1063–1075. (10.1016/j.jhep.2018.01.019) 29408694 PMC5893377

[bib34] Johnson RK Appel LJ Brands M Howard BV Lefevre M Lustig RH Sacks F Steffen LM Wylie-Rosett J & American Heart Association Nutrition Committee of the Council on Nutrition, Physical Activity, and Metabolism and the Council on Epidemiology and Prevention 2009 Dietary sugars intake and cardiovascular health: a scientific statement from the American Heart Association. Circulation 120 1011–1020. (10.1161/CIRCULATIONAHA.109.192627) 19704096

[bib35] Kay Parker M 2010 High fructose corn syrup: production, uses and public health concerns SaVCN. Biotechnology and Molecular Biology Reviews 5 71–78. (10.5897/BMBR2010.0009)

[bib36] Kazierad DJ Chidsey K Somayaji VR Bergman AJ Birnbaum MJ Calle RA 2021 Inhibition of ketohexokinase in adults with NAFLD reduces liver fat and inflammatory markers: a randomized phase 2 trial. Med 2 800–813.e3. (10.1016/j.medj.2021.04.007) 35590219

[bib37] Kim MS Krawczyk SA Doridot L Fowler AJ Wang JX Trauger SA Noh HL Kang HJ Meissen JK Blatnik M 2016 ChREBP regulates fructose-induced glucose production independently of insulin signaling. Journal of Clinical Investigation 126 4372–4386. (10.1172/JCI81993) 27669460 PMC5096918

[bib38] Kmietowicz Z 2012 Countries that use large amounts of high fructose corn syrup have higher rates of type 2 diabetes. BMJ 345 e7994. (10.1136/bmj.e7994) 23187791

[bib39] Koepsell H 2020 Glucose transporters in the small intestine in health and disease. Pflugers Archiv 472 1207–1248. (10.1007/s00424-020-02439-5) 32829466 PMC7462918

[bib40] Kohjima M Enjoji M Higuchi N Kato M Kotoh K Yoshimoto T Fujino T Yada M Yada R Harada N 2007 Re-evaluation of fatty acid metabolism-related gene expression in nonalcoholic fatty liver disease. International Journal of Molecular Medicine 20 351–358. (10.3892/ijmm.20.3.351) 17671740

[bib41] Koo HY Miyashita M Simon Cho BH Nakamura MT 2009 Replacing dietary glucose with fructose increases ChREBP activity and SREBP-1 protein in rat liver nucleus. Biochemical and Biophysical Research Communications 390 285–289. (10.1016/j.bbrc.2009.09.109) 19799862

[bib42] Korbecki J Bajdak-Rusinek K 2019 The effect of palmitic acid on inflammatory response in macrophages: an overview of molecular mechanisms. Inflammation Research 68 915–932. (10.1007/s00011-019-01273-5) 31363792 PMC6813288

[bib43] Lambert JE Ramos-Roman MA Browning JD Parks EJ 2014 Increased de novo lipogenesis is a distinct characteristic of individuals with nonalcoholic fatty liver disease. Gastroenterology 146 726–735. (10.1053/j.gastro.2013.11.049) 24316260 PMC6276362

[bib44] Lanaspa MA Andres-Hernando A Orlicky DJ Cicerchi C Jang C Li N Milagres T Kuwabara M Wempe MF Rabinowitz JD 2018 Ketohexokinase C blockade ameliorates fructose-induced metabolic dysfunction in fructose-sensitive mice. Journal of Clinical Investigation 128 2226–2238. (10.1172/JCI94427) 29533924 PMC5983342

[bib45] Lecoultre V Egli L Carrel G Theytaz F Kreis R Schneiter P Boss A Zwygart K Lê KA Bortolotti M 2013 Effects of fructose and glucose overfeeding on hepatic insulin sensitivity and intrahepatic lipids in healthy humans. Obesity 21 782–785. (10.1002/oby.20377) 23512506

[bib46] Lim JS Mietus-Snyder M Valente A Schwarz JM Lustig RH 2010 The role of fructose in the pathogenesis of NAFLD and the metabolic syndrome. Nature Reviews. Gastroenterology and Hepatology 7 251–264. (10.1038/nrgastro.2010.41) 20368739

[bib47] Linden AG Li S Choi HY Fang F Fukasawa M Uyeda K Hammer RE Horton JD Engelking LJ Liang G 2018 Interplay between ChREBP and SREBP-1c coordinates postprandial glycolysis and lipogenesis in livers of mice. Journal of Lipid Research 59 475–487. (10.1194/jlr.M081836) 29335275 PMC5832931

[bib48] Löwik MRH 2021 Assessment and evaluation of the intake of sugars in European countries. Applied Sciences 11 11983. (10.3390/app112411983)

[bib49] Ma J Karlsen MC Chung M Jacques PF Saltzman E Smith CE Fox CS McKeown NM 2016 Potential link between excess added sugar intake and ectopic fat: a systematic review of randomized controlled trials. Nutrition Reviews 74 18–32. (10.1093/nutrit/nuv047) 26518034 PMC4859325

[bib50] Maedler K Oberholzer J Bucher P Spinas GA Donath MY 2003 Monounsaturated fatty acids prevent the deleterious effects of palmitate and high glucose on human pancreatic β-cell turnover and function. Diabetes 52 726–733. (10.2337/diabetes.52.3.726) 12606514

[bib51] Maedler K Spinas GA Dyntar D Moritz W Kaiser N Donath MY 2001 Distinct effects of saturated and monounsaturated fatty acids on beta-cell turnover and function. Diabetes 50 69–76. (10.2337/diabetes.50.1.69) 11147797

[bib52] Maersk M Belza A Stødkilde-Jørgensen H Ringgaard S Chabanova E Thomsen H Pedersen SB Astrup A Richelsen B 2012 Sucrose-sweetened beverages increase fat storage in the liver, muscle, and visceral fat depot: a 6-mo randomized intervention study. American Journal of Clinical Nutrition 95 283–289. (10.3945/ajcn.111.022533) 22205311

[bib53] Malik VS Hu FB 2022 The role of sugar-sweetened beverages in the global epidemics of obesity and chronic diseases. Nature Reviews. Endocrinology 18 205–218. (10.1038/s41574-021-00627-6) PMC8778490 35064240

[bib54] Maruhama Y Macdonald I 1973 Incorporation of orally administered glucose-U-14C and fructose-U-14C into the triglyceride of liver, plasma, and adipose tissue of rats. Metabolism: Clinical and Experimental 22 1205–1215. (10.1016/0026-0495(7390208-4) 4726370

[bib55] McGarry JD Mannaerts GP Foster DW 1977 A possible role for malonyl-CoA in the regulation of hepatic fatty acid oxidation and ketogenesis. Journal of Clinical Investigation 60 265–270. (10.1172/JCI108764) 874089 PMC372365

[bib56] Mendeloff AI Weichselbaum TE 1953 Role of the human liver in the assimilation of intravenously administered fructose. Metabolism: Clinical and Experimental 2 450–458. 13110753

[bib57] Moore JB 2010 Non-alcoholic fatty liver disease: the hepatic consequence of obesity and the metabolic syndrome. Proceedings of the Nutrition Society 69 211–220. (10.1017/S0029665110000030) 20158939

[bib58] Moskowitz HR 1970 Ratio scales of sugar sweetness. Perception and Psychophysics 7 315–320. (10.3758/BF03210175)

[bib59] Nagai Y Nishio Y Nakamura T Maegawa H Kikkawa R Kashiwagi A 2002 Amelioration of high fructose-induced metabolic derangements by activation of PPARalpha. American Journal of Physiology. Endocrinology and Metabolism 282 E1180–E1190. (10.1152/ajpendo.00471.2001) 11934685

[bib60] Noubiap JJ Nansseu JR Lontchi-Yimagou E Nkeck JR Nyaga UF Ngouo AT Tounouga DN Tianyi FL Foka AJ Ndoadoumgue AL 2022 Global, regional, and country estimates of metabolic syndrome burden in children and adolescents in 2020: a systematic review and modelling analysis. Lancet. Child and Adolescent Health 6 158–170. (10.1016/S2352-4642(2100374-6) 35051409

[bib62] Ouyang X Cirillo P Sautin Y McCall S Bruchette JL Diehl AM Johnson RJ Abdelmalek MF 2008 Fructose consumption as a risk factor for non-alcoholic fatty liver disease. Journal of Hepatology 48 993–999. (10.1016/j.jhep.2008.02.011) 18395287 PMC2423467

[bib63] Parks EJ Skokan LE Timlin MT Dingfelder CS 2008 Dietary sugars stimulate fatty acid synthesis in adults. Journal of Nutrition 138 1039–1046. (10.1093/jn/138.6.1039) 18492831 PMC2546703

[bib64] Patel C Douard V Yu S Tharabenjasin P Gao N & Ferraris RP 2015a Fructose-induced increases in expression of intestinal fructolytic and gluconeogenic genes are regulated by GLUT5 and KHK. American Journal of Physiology. Regulatory, Integrative and Comparative Physiology 309 R499–R509. (10.1152/ajpregu.00128.2015) 26084694 PMC4591376

[bib65] Patel C Sugimoto K Douard V Shah A Inui H Yamanouchi T & Ferraris RP 2015b Effect of dietary fructose on portal and systemic serum fructose levels in rats and in KHK-/- and GLUT5-/- mice. American Journal of Physiology. Gastrointestinal and Liver Physiology 309 G779–G790. (10.1152/ajpgi.00188.2015) 26316589 PMC4628968

[bib66] Prager GN Ontko JA 1976 Direct effects of fructose metabolism on fatty acid oxidation in a recombined rat liver mitochondria-high speed supernatant system. Biochimica et Biophysica Acta 424 386–395. (10.1016/0005-2760(7690028-x) 1259967

[bib67] Riazi K Azhari H Charette JH Underwood FE King JA Afshar EE Swain MG Congly SE Kaplan GG Shaheen AA 2022 The prevalence and incidence of NAFLD worldwide: a systematic review and meta-analysis. Lancet. Gastroenterology and Hepatology 7 851–861. (10.1016/S2468-1253(2200165-0) 35798021

[bib68] Rotter V Nagaev I Smith U 2003 Interleukin-6 (IL-6) induces insulin resistance in 3T3-L1 adipocytes and is, like IL-8 and tumor necrosis factor-alpha, overexpressed in human fat cells from insulin-resistant subjects. Journal of Biological Chemistry 278 45777–45784. (10.1074/jbc.M301977200) 12952969

[bib69] Rumessen JJ Gudmand-Høyer E 1986 Absorption capacity of fructose in healthy adults. Comparison with sucrose and its constituent monosaccharides. Gut 27 1161–1168. (10.1136/gut.27.10.1161) 3781328 PMC1433856

[bib70] Sahota AK Shapiro WL Newton KP Kim ST Chung J & Schwimmer JB 2020 Incidence of nonalcoholic fatty liver disease in children: 2009–2018. Pediatrics 146 e20200771. (10.1542/peds.2020-0771) PMC7706110 33214329

[bib71] Saklayen MG 2018 The global epidemic of the metabolic syndrome. Current Hypertension Reports 20 12. (10.1007/s11906-018-0812-z) PMC5866840 29480368

[bib72] Schwarz JM Noworolski SM Erkin-Cakmak A Korn NJ Wen MJ Tai VW Jones GM Palii SP Velasco-Alin M Pan K 2017 Effects of dietary fructose restriction on liver fat, De Novo Lipogenesis, and Insulin Kinetics in Children With Obesity. Gastroenterology 153 743–752. (10.1053/j.gastro.2017.05.043) 28579536 PMC5813289

[bib73] Schwarz JM Noworolski SM Wen MJ Dyachenko A Prior JL Weinberg ME Herraiz LA Tai VW Bergeron N Bersot TP 2015 Effect of a high-fructose weight-maintaining diet on lipogenesis and liver fat. Journal of Clinical Endocrinology and Metabolism 100 2434–2442. (10.1210/jc.2014-3678) 25825943 PMC4454806

[bib74] Sekkarie A Welsh JA Northstone K Stein AD Ramakrishnan U Vos MB 2021 Associations between free sugar and sugary beverage intake in early childhood and adult NAFLD in a population-based UK cohort. Children (Basel) 8. (10.3390/children8040290) PMC8068295 33917875

[bib75] Serna-Saldivar SO 2016 Maize: foods from maize. In Reference Module in Food Science [epub]. (10.1016/B978-0-08-100596-5.00126-8)

[bib76] Singh GM Micha R Khatibzadeh S Shi P Lim S Andrews KG Engell RE Ezzati M Mozaffarian D & Global Burden of Diseases Nutrition and Chronic Diseases Expert Group (NutriCoDE) 2015 Global, regional, and national consumption of sugar-sweetened beverages, fruit juices, and milk: a systematic assessment of beverage intake in 187 countries. PLoS One 10 e0124845. (10.1371/journal.pone.0124845) PMC4526649 26244332

[bib77] Smith GI Shankaran M Yoshino M Schweitzer GG Chondronikola M Beals JW Okunade AL Patterson BW Nyangau E Field T 2020 Insulin resistance drives hepatic de novo lipogenesis in nonalcoholic fatty liver disease. Journal of Clinical Investigation 130 1453–1460. (10.1172/JCI134165) 31805015 PMC7269561

[bib78] Softic S Cohen DE Kahn CR 2016 Role of dietary fructose and hepatic de novo lipogenesis in fatty liver disease. Digestive Diseases and Sciences 61 1282–1293. (10.1007/s10620-016-4054-0) 26856717 PMC4838515

[bib79] Softic S Gupta MK Wang GX Fujisaka S O'Neill BT Rao TN Willoughby J Harbison C Fitzgerald K Ilkayeva O 2017 Divergent effects of glucose and fructose on hepatic lipogenesis and insulin signaling. Journal of Clinical Investigation 127 4059–4074. (10.1172/JCI94585) 28972537 PMC5663363

[bib80] Softic S Meyer JG Wang GX Gupta MK Batista TM Lauritzen HPMM Fujisaka S Serra D Herrero L Willoughby J 2019 Dietary sugars alter hepatic fatty acid oxidation via transcriptional and post-translational modifications of mitochondrial proteins. Cell Metabolism 30 735–753.e4. (10.1016/j.cmet.2019.09.003) 31577934 PMC7816129

[bib81] Song Z Xiaoli AM & Yang F 2018 Regulation and metabolic significance of de novo lipogenesis in adipose tissues. Nutrients 10 1383. (10.3390/nu10101383) PMC6213738 30274245

[bib82] Staiger H Staiger K Stefan N Wahl HG Machicao F Kellerer M Häring HU 2004 Palmitate-induced interleukin-6 expression in human coronary artery endothelial cells. Diabetes 53 3209–3216. (10.2337/diabetes.53.12.3209) 15561952

[bib83] Stanhope KL Schwarz JM Keim NL Griffen SC Bremer AA Graham JL Hatcher B Cox CL Dyachenko A Zhang W 2009 Consuming fructose-sweetened, not glucose-sweetened, beverages increases visceral adiposity and lipids and decreases insulin sensitivity in overweight/obese humans. Journal of Clinical Investigation 119 1322–1334. (10.1172/JCI37385) 19381015 PMC2673878

[bib84] Tappy L Lê KA 2010 Metabolic effects of fructose and the worldwide increase in obesity. Physiological Reviews 90 23–46. (10.1152/physrev.00019.2009) 20086073

[bib85] Taylor SR Ramsamooj S Liang RJ Katti A Pozovskiy R Vasan N Hwang SK Nahiyaan N Francoeur NJ Schatoff EM 2021 Dietary fructose improves intestinal cell survival and nutrient absorption. Nature 597 263–267. (10.1038/s41586-021-03827-2) 34408323 PMC8686685

[bib86] Te Morenga LA Howatson AJ Jones RM Mann J 2014 Dietary sugars and cardiometabolic risk: systematic review and meta-analyses of randomized controlled trials of the effects on blood pressure and lipids. American Journal of Clinical Nutrition 100 65–79. (10.3945/ajcn.113.081521) 24808490

[bib87] Testa R Olivieri F Bonfigli AR Sirolla C Boemi M Marchegiani F Marra M Cenerelli S Antonicelli R Dolci A 2006 Interleukin-6-174 G > C polymorphism affects the association between IL-6 plasma levels and insulin resistance in type 2 diabetic patients. Diabetes Research and Clinical Practice 71 299–305. (10.1016/j.diabres.2005.07.007) 16140413

[bib88] Thorens B Mueckler M 2010 Glucose transporters in the 21st Century. American Journal of Physiology. Endocrinology and Metabolism 298 E141–E145. (10.1152/ajpendo.00712.2009) 20009031 PMC2822486

[bib89] Vessby B 2003 Dietary fat, fatty acid composition in plasma and the metabolic syndrome. Current Opinion in Lipidology 14 15–19. (10.1097/00041433-200302000-00004) 12544656

[bib90] Wang X Sato R Brown MS Hua X Goldstein JL 1994 SREBP-1, a membrane-bound transcription factor released by sterol-regulated proteolysis. Cell 77 53–62. (10.1016/0092-8674(9490234-8) 8156598

[bib91] Weigert C Brodbeck K Staiger H Kausch C Machicao F Häring HU Schleicher ED 2004 Palmitate, but not unsaturated fatty acids, induces the expression of interleukin-6 in human myotubes through proteasome-dependent activation of nuclear factor-kappaB. Journal of Biological Chemistry 279 23942–23952. (10.1074/jbc.M312692200) 15028733

[bib61] WHO 2015 Guideline: sugars intake for adults and children. Geneva, Switzerland: WHO. (available at: https://www.who.int/publications/i/item/9789241549028) 25905159

[bib92] Yamashita H Takenoshita M Sakurai M Bruick RK Henzel WJ Shillinglaw W Arnot D & Uyeda K 2001 A glucose-responsive transcription factor that regulates carbohydrate metabolism in the liver. PNAS 98 9116–9121. (10.1073/pnas.161284298) 11470916 PMC55382

[bib93] Yin J Zhu Y Malik V Li X Peng X Zhang FF Shan Z Liu L 2021 Intake of sugar-sweetened and low-calorie sweetened beverages and risk of cardiovascular disease: a meta-analysis and systematic review. Advances in Nutrition 12 89–101. (10.1093/advances/nmaa084) 32696948 PMC7850046

[bib94] Zhao S Jang C Liu J Uehara K Gilbert M Izzo L Zeng X Trefely S Fernandez S Carrer A 2020 Dietary fructose feeds hepatic lipogenesis via microbiota-derived acetate. Nature 579 586–591. (10.1038/s41586-020-2101-7) 32214246 PMC7416516

